# The Effect of Age on High-Dose Therapy with Autologous Stem Cell Support in Multiple Myeloma: A Single-Center Experience

**DOI:** 10.3390/jcm13144142

**Published:** 2024-07-16

**Authors:** Elcin Erdogan Yucel, Ayse Tugce Kirmaz, Merve Kakci, Aylin Fatma Yavuz, Tugce Sencelikel, Inci Alacacioglu, Guner Hayri Ozsan

**Affiliations:** 1 Department of Hematology, University of Health Science, Izmir City Hospital, 35180 Izmir, Turkey; 2 Department of Internal Medicine, Dokuz Eylul University, 35220 Izmir, Turkey; aysetugce.kirmaz@deu.edu.tr; 3 Department of Hematology, Dokuz Eylul University, 35220 Izmir, Turkey; merve.kakci@deu.edu.tr (M.K.); aylinfatma.karatas@deu.edu.tr (A.F.Y.); inci.alacacioglu@deu.edu.tr (I.A.); hayri.ozsan@deu.edu.tr (G.H.O.); 4 Department of Biostatistics, Medipol University, Ankara 34810, Turkey; tugce.sencelikel@ankaramedipol.edu.tr

**Keywords:** multiple myeloma, stem cell transplantation, age

## Abstract

**Background:** This retrospective one-center study demonstrates the complications related to high-dose therapy with autologous stem cell support (HDT) and the survival of multiple myeloma (MM) patients according to age groups. **Methods**: We categorized the patients into two groups: those who were ≤65 years old (group 1) (*N* = 115) and those who were >65 years old (group 2) (*N* = 26). The mean duration of follow-up was 48 (1–125) months. **Results:** In group 2 patients, the use of a reduced dosage of melphalan (12 [46%] versus 30 [26%]) was more frequent in comparison to group 1 (*p* = 0.046). There was a statistically significant difference between the two groups regarding the neutrophil engraftment days (*p* = 0.001) and the median progression-free survival (PFS) (*p* = 0.02). The PFS was 44 months for group 1 and 30 months for group 2. There was no statistically significant difference between the groups in relation to the median duration of hospitalization, presence of bacteremia, intravenous antibiotic administration, and overall survival (OS). **Conclusions:** The study’s results indicate that HDT is a reliable method of treatment for older patients with MM, provided that they obtain a suitable conditioning regimen and, furthermore, these patients achieved a comparable OS rate to that of younger patients.

## 1. Introduction 

Multiple myeloma (MM) is a disease that typically occurs in advanced age individuals and originates from plasma cells. With the average age of society on the rise, the frequency of diagnosis is also on the upswing. Utilizing high-dose therapy with autologous stem cell support (HDT) as consolidation in the treatment of newly diagnosed MM patients still remains the standard treatment approach [1,2]. In studies examining the impact of HDT on survival, the generally accepted upper limit was age 65. However, the situation may differ between different countries and clinics. It is worth noting that transplantation procedures can still be performed on patients who are 70 years old or older. When comparing patients aged 65–70 years with younger patients in retrospective analyses, it was found that there were no significant differences in progression-free (PFS), overall survival (OS), and transplant-related mortality between age groups [3,4]. 

Typically, these studies involve patients who are fit. Various factors, including the increased prevalence of comorbidities in older MM patients, patient fragility, and the advancements in new-generation treatments, impact the clinician’s decision regarding transplantation. The retrospective analysis of the Gimema study group, which comprised patients over the age of 70, compared the statistics of 12 patients who had transplantation to 97 patients who did not. PFS and OS were higher in the transplant group (56.4 vs. 26.1 months; 107.6 vs. 46.5). This and similar studies have indicated that transplanting at an older age improves survival rates [2,5].

In this study, we examined the results of HDT in patients with MM by grouping them as under and above 65 years of age. The objective of this study was to examine the dependability of HDT and its impact on mortality in older patients who are over the age of 65.

## 2. Methods 

### 2.1. Study Design and Patients

Between January 2012 and January 2022, we retrospectively analyzed the data of patients diagnosed with multiple myeloma (MM) who had symptomatic, measurable disease according to the criteria set by the International Myeloma Working Group (IMWG). The study included patients aged between 18 and 75 years who underwent HDT within 12 months of diagnosis at our center. The study was executed in compliance with the principles outlined in the Declaration of Helsinki. The study excluded patients with plasma cell leukemia, amyloidosis, relapsed multiple myeloma, patients who had undergone previous autologous or allogeneic transplantation, patients with a secondary malignancy, those who had received high-dose chemotherapy, and those who saw disease progression after 4–6 cycles of induction. The study included patients who met the following criteria: eligibility for HDT, ejection fraction more than 40% without symptomatic cardiac disease, and carbon monoxide diffusion capacity greater than 50% without symptomatic pulmonary disease.

In the study, a total of 141 patients were ultimately enrolled. From the patient files, information on demographic features, myeloma chain type, presence of end organ damage, disease stages, transplantation date, and engraftment days, as well as survival and relapse data, were documented. The patients were categorized into two groups: those who were ≤65 years (group 1) and those who were >65 years (group 2). The groups were evaluated based on age, gender, monoclonal protein type, disease stage, comorbidities, existence of end organ damage (such as anemia, hypercalcemia, lytic lesions, renal disorder), presence of plasmacytoma, and dosage of melphalan employed in the conditioning regimen. The study examined the engraftment days following HDT, the occurrence of bacteremia, the duration of hospitalization, hematological results, and the risk factors that impact survival. The international staging system (ISS) and revised ISS were utilized for risk scoring. 

### 2.2. Induction Chemotherapy and Stem Cell Mobilization

The primary induction treatments administered to the patients consisted of 3–6 cycles of cyclophosphamide–bortezomib–dexamethasone (VCD), vincristine–adriablastin dexamethasone (VAD), bortezomib–dexamethasone (Vd), and bortezomib–lenalidomide dexamethasone (VRd).

### 2.3. Stem Cell Mobilization and Preparation Regimen for Transplantation

The mobilization process involved the administration of subcutaneous granulocyte colony-stimulating factor (GCSF) (10 μg/kg/day). GCSF was administered either as a single therapy or in combination with cyclophosphamide (1.5 g/m^2^ for 2 consecutive days). The goal was to mobilize 3 × 10^6^ CD34+ cells/kg, and this goal was successfully reached in 97.8% of patients. The administration of Melphalan in the conditioning regimen involved two different doses: a low dosage of 140 mg/m^2^ and a standard dose of 200 mg/m^2^. The selection of the dose was based on the patients’ physical performance, comorbidities, and renal functions. 

### 2.4. Evaluation of Engraftment and Toxicity

Neutrophil engraftment was considered achieved when the absolute neutrophil count reached or exceeded 0.5 × 10^9^ /L for three consecutive days. Platelet engraftment, on the other hand, was considered achieved when platelet levels reached or exceeded 20 × 10^9^ /L without requiring replacement.

### 2.5. Statistical Analysis

The suitability of the variables for normal distribution was assessed using the Kolmogorov–Smirnov test. The association between two category variables was examined using either the Chi-square test or Fischer’s exact test. The Mann–Whitney U Test was used to compare two independent means. Survival analysis was conducted to determine overall survival using the Kaplan–Meier log-rank test. The Cox proportional hazards regression model was utilized to perform multivariate analysis for PFS and OS. The risk was quantified using hazard ratio (HR) along with a 95% confidence interval (CI). The data analysis was performed using Statistical Package for the Social Science (SPSS Inc, Chicago, IL, USA) version 24.0, and a *p*-value of <0.05 was considered to be statistically significant.

## 3. Results

The mean duration of follow-up was 48 (1–125) months. Group 1 consisted of 115 patients, whereas group 2 had 26 individuals. The patients had a median age of 58 (26–72) years. In group 1, the median age was 56 (26–65). In group 2, the median age was 67 (66–72). The research comprised 88 male patients and 53 female patients, resulting in a male-to-female ratio of 88:53. There was no significant difference observed between group 1 and group 2 regarding the monoclonal protein type, ISS and R-ISS stages, creatine value at diagnosis, anemia, presence of lytic lesion, the hematopoetic cell transplantation-specific comorbidity index (HCT-CI) scores and serum calcium levels (Table 1).

Out of the 141 patients participating in the study, 42 (30%) received a reduced dose of melphalan (140 mg/m^2^) as a conditioning regimen. In group 2 patients, the use of a reduced dosage of melphalan (12 [46%] versus 30 [26%]) was more frequent in comparison to group 1. There was a statistically significant difference (*p* = 0.046) (Table 1).

The median days for platelet engraftment in group 1 and group 2 were 12 (8–27) and 12 (8–20) days, respectively. There was no statistically significant difference between the two groups (*p* = 0.3). In group 1, the median time for neutrophil engraftment was 10 days (7–22), whereas in group 2, it was 12 days (9–16). This difference was statistically significant (*p* = 0.001) (Table 1). There was no statistically significant difference between the neutrophil engraftment day and the infused CD34+ stem cell value (*p* = 0.07).

**Figure 1:**
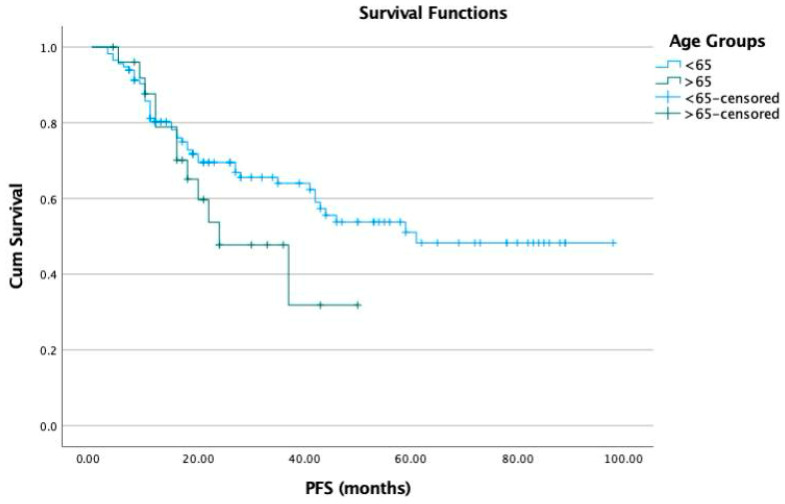
Progression-free survival (PFS) according to the age groups.

**Figure 2:**
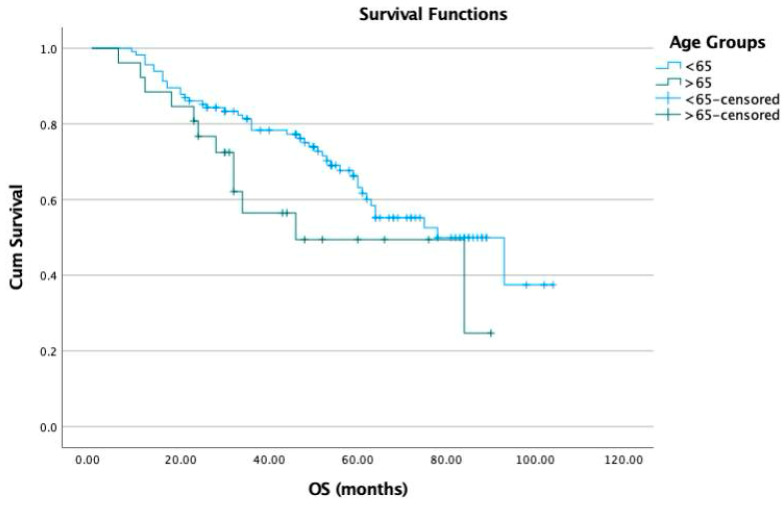
Overall survival (OS) according to the age groups.

The Kaplan–Meier analysis revealed that the median progression-free survival (PFS) was 44 months for group 1 and 30 months for group 2. The PFS in group 2 was statistically significantly lower than in group 1 (*p* = 0.02) (Figure 1). The median OS was 78 months in group 1 and 67 months in group 2 (Figure 2). There was no statistically significant difference between the groups in terms of OS (*p* = 0.1).

During the univariate analysis conducted on patients in Group 1, only hypercalcemia (HR: 2.82 95% CI: 1.42–5.58, *p* = 0.003) was identified as an independent prognostic factor for PFS. In our multivariate analysis of group 1, anemia (HR: 2.2, 95% CI: 1.01–4.84, *p* = 0.046) and the presence of plasmacytoma (HR: 3.0, 95% CI: 1.24–7.43, *p* = 0.015) were identified as independent prognostic factors for PFS (Table 2). 

In terms of conditioning regimen, 73% of patients in group 1 preferred a dosage of 200 mg/m^2^ melphalan, whereas 53% of patients in group 2 received the same dosage (*p* = 0.046). In group 2, the administration of a reduced dosage (140 mg/m^2^) of melphalan was preferred more often compared to group 1 (Table 1).

Lenalidomide maintenance was given to 12 high-risk patients who underwent HCT. Three of these patients were in group 2 and nine in group 1. Lenalidomide maintenance was stopped before the 24th month in two patients, aged 68 and 64, due to cytopenia and infection and in five patients due to progression.

The median duration of hospitalization was 22 (13–64) days. There was no statistically significant difference seen between the groups in relation to the median duration of hospitalization. Bacteremia and intravenous antibiotic administration were necessary after transplantation in 105 patients (75%). No significant difference was seen between the groups (Table 1). In the multivariate analysis conducted on all patients, albumin was identified as an independent prognostic factor for PFS (HR: 0.57, 95% CI: 0.34–0.96, *p* = 0.035) and OS (HR: 0.48, 95% CI: 0.29–0.79, *p* = 0.004). Additionally, the ISS stage was also found to be an independent prognostic factor for both PFS (HR: 0.4, 95% CI: 0.18–0.89, *p* = 0.025) and OS (HR: 0.38, 95% CI: 0.16–0.91, *p* = 0.025) (Table 3). 

## 4. Discussion

Despite new treatment modalities for older MM patients, HDT remains one of the first-line treatment options. In our study, we discovered that ISS stages at the time of diagnosis had a comparable distribution in the groups under and over 65 years old. We found no significant difference between age groups in the presence of osteolytic lesions and plasmacytomas. According to studies published by the IMWG and IFM, it was stated that young patients were more often in the ISS1 stage at the time of diagnosis [6,7]. In a study performed in Uruguay, Bove et al. attributed this scenario to a high albumin level. Again, the study found that extramedullary findings, plasmacytoma, and osteolytic lesions were more common in younger patient groups [8]. When we examined the age groupings in our study, we discovered that 46.1% of patients over 65 years old were diagnosed with ISS stage 1 at the time of diagnosis, whereas 30.4% of patients aged 65 and under were diagnosed with ISS stage 1. As a result, we found that elderly patients were diagnosed earlier. We believe this is connected to the earlier use of the diagnostic algorithm in older patients and the quicker diagnosis of elderly patients under our country’s conditions.

In the Myeloma XI study, PFS was observed to decrease with increasing age. The study found that the median PFS was 50.8 months for those under the age of 65, 40 months for those aged 65–69, and 34.4 months for those aged 70–75. Although there was an apparent difference between the patient groups aged over 70 and those under 65, the difference did not reach statistical significance in patients aged over 70 and between 65 and 69 years old [9]. For our study, we categorized the patients based on their age, specifically distinguishing between those who were under and over 65 years of age, considering these data.

Our study revealed a statistically significant decrease in PFS among patients aged 65 and above compared to those under 65. No difference was noted for OS. In their study on 132 transplanted MM patients, Marini et al. found no significant correlation between age and OS [10]. Subsequently, Schain et al. shared the data of 1479 patients with MM who underwent HDT in a letter [11]. They reported that the time to the next treatment and OS were significantly higher in the younger patients compared to the older patients. They demonstrated this effect, especially in individuals who received treatment before 2013, and indicated that the correlation between age and OS diminished after 2013. In this study, we enrolled patients who had treatment from 2012 to 2022. 

This is a period where new treatments are used in myeloma patients, such as monoclonal antibodies and immunomodulators, and there are extensive treatment options for both maintenance and post-progression therapy following HDT. In a phase III, double-blind, placebo-controlled TOURMALINE-MM4 study, 706 MM patients who were newly diagnosed and were not suitable for autologous transplantation were evaluated for ixazomib maintenance after induction therapy. The overall median age of these patients was 73 years, and the median PFS was 17.4 months with ixazomib maintenance, while it was 9.4 months with placebo. This study showed that PFS could be improved with the maintenance of the proteasome inhibitor ixazomib in non-transplant MM patients [12]. In the Phase III MAIA study, an increase in OS and PFS was detected when daratumumab was added to lenalidomide and dexamethasone treatment in transplantation-ineligible MM patients [13]. Furthermore, these treatments had a positive effect on the survival of patients who were unable to undergo cytotoxic treatment due to their advanced age. In our study, we aimed to evaluate the age factor in transplanted patients. We think that prospective studies and meta-analyses that compare novel treatment methods and HDT in elderly patients are needed. In this context, we think that our study will contribute to the literature. 

We discovered that lower dosage melphalan was utilized more frequently as a preparation regimen in patients over 65 years old than in patients 65 and under. Similar results were found in the Myeloma XI study [9]. Furthermore, in the Myeloma XI study, patients receiving a reduced dosage of melphalan had a lower PFS than those receiving a higher dose. The authors ascribed the reduced PFS in patients receiving low-dose melphalan to aging rather than the melphalan dosage [9]. In a prospective study, 114 patients treated with high doses of melphalan were evaluated for post-treatment area under the concentration vs. time curve (AUC). They found that above-average AUC values were associated with improvement in OS but did not make a difference in PFS [14]. In this study, we found no significant association between melphalan dosage and PFS. As a result, similar to the myeloma XI study, we believe that PFS is associated with age and a high incidence of comorbidities rather than melphalan dosage.

Although the times of platelet engraftment were similar in-patient groups aged under and over 65 years, we observed that the process of neutrophil engraftment was statistically significantly much longer in patients over 65 years of age (*p* < 0.001) (Table 1). In 2022, Fedorov et al. classified MM and lymphoma patients who had HDT according to their age. They discovered that neutrophil and platelet engraftment was significantly longer in patients over 75 years of age compared to those aged 55–65 years [15]. Both our study and Federov’s study found that increasing the engraftment times did not have a significant impact on hospitalization or the necessity for replacement. We believe this is related to transplanted patients’ relatively high-performance status and capacity to access supportive treatments as needed.

We recognize that our study has some limitations. The first limitation of our study is its retrospective nature. Another limitation is the low patient population and short follow-up period of 48 months. Furthermore, because the patients’ chromosomal mutations were unclear, R-ISS analysis could not be performed. Statistical evaluation could not be made due to heterogeneity in induction regimens and the small number of patients receiving maintenance therapy. Our study does not determine whether HDT improves PFS or OS compared to not receiving HDT for patients aged 65 to 75. There is a need for prospective studies in this context that consider the use of upfront daratumumab with immunomodulatory drugs (IMiDs) in older MM patients.

## 5. Conclusions

The study’s results indicate that HDT is a reliable method of treatment for older patients with MM, provided that they receive the suitable conditioning regimen and necessary supporting treatments; furthermore, these patients achieved a comparable OS rate to that of younger patients. There is a need for prospective, multicenter studies involving a greater number of patients in order to provide greater clarity on this matter.

## Figures and Tables

**Table 1:** Patient characteristics of the study population.

Variables	Overall (*n* = 141)	Group 1 (*n* = 115)	Group 2 (*n* = 26)	*p* Value
Age (years)	58 (26–72)	56 (26–65)	67.5 (66–72)	<0.001 ^a^
Gender (*n*, %)				0.582 ^b^
Male	88 (62.4)	73 (63.5)	15 (57.7)	
Female	53 (37.5)	42 (36.5)	11 (42.5)	
Immunglobulin Type (*n*, %)				0.081 ^b^
G	85 (60.3)	74 (64.3)	11 (42.3)	
A	29 (20.6)	20 (17.4)	9 (34.6)	
Others	27 (19.1)	21 (18.3)	6 (23.1)	
Multiple plasmacytoma (*n*,%)				0.578 ^b^
Yes	29 (20.5)	22 (19.1)	7 (26.9)	
No	112 (79.5)	93 (65.9)	19 (73.1)	
ISS at diagnosis (*n*,%)				0.182 ^b^
ISS 1	43 (30.4)	35 (30.4)	12 (46.1)	
ISS 2	46 (32.6)	36 (31.3)	9 (34.6)	
ISS 3	48 (34)	41 (35.6)	4 (15.3)	
Unknown	4 (2.8)	3 (2.6)	1 (3.8)	
R-ISS at diagnosis (*n*,%)				>0.999 ^c^
R-ISS 1	16 (11.3)	14 (12.2)	2 (7.6)	
R-ISS 2	34 (24.2)	28(24.4)	6 (23.2)	
R-ISS 3	23 (16.3)	18 (15.6)	5 (19.2)	
Unknown	68 (48.2)	55 (47.8)	13 (50)	
Calcium (mg/dL)				0.071 ^c^
>11	18 (12.7)	11 (9.5)	7 (26.9)	
≤11	123 (87.3)	104 (90.5)	19 (73.1)	
Litic lesions (*n*, %)				0.891 ^b^
Yes	104 (73.7)	85 (73.9)	19 (73)	
No	37 (26.3)	30 (26.1)	7 (27)	
Hemoglobin (g/dL) (*n*, %)				0.099 ^b^
<10	56 (39.7)	48 (41.7)	8 (30.7)	
≥10	85 (60.2)	67 (58.3)	18 (69.3)	
Creatinine (mg/dL) (*n*, %)				0.086 ^b^
≥2	28 (19.8)	25 (21.7)	3 (11.5)	
<2	113 (80.2)	90 (78.3)	23 (88.5)	
Plazmacytoma				0.375 ^b^
Yes	29 (20.5)	22 (19.1)	7 (26.9)	
No	112 (81.5)	93 (80.9)	19 (73.1)	
Conditioning (mg/m^2^)				0.046 ^b^
Melphalan 140	42 (29.8)	30 (26.1)	12 (46.2)	
Melphalan 200	99 (70.2)	85 (73.9)	14 (53.8)	
Time of Engraftment (days)				
Platelet ≥ 20 × 10^3^/μL	12 (8–27)	12 (8–27)	12 (8–20)	0.387 ^a^
Neutrophil ≥ 0.5 × 10^3^/μL	10 (7–22)	10 (7–22)	12 (9–16)	<0.001 ^a^
Length of Hospitalization (days)	22 (13–64)	21 (13–64)	23.5 (16–33)	0.090 ^a^
Bacteremia (*n*, %)				0.182 b ^a^
Yes	105 (75.5)	88 (77.9)	17 (65.4)	
No	36 (24.5)	27 (22.1)	9 (34.6)	
Infused CD34^+^ cells (10^6^/kg) (mean ± Sd)	6.24 ± 2.4	6.36 ± 2.5	5.7 ± 1.8	0.170 ^a^
HCT-CI score n (%)				0.12 ^b^
0–2	60 (42)	52 (45)	8 (30)	
≥3	81 (58)	63 (55)	18 (70)	

^a^: Mann–Whitney U test; Medyan (Minimum–Maximum), ^b^: Pearson Chi-square test; n (%), ^c^: Fisher–Freeman–Halton Exact Test; n (%), ISS: international staging system, R-ISS: revise international staging system, HCT:CI: The hematopoetic cell transplantation-specific comorbidity index.

**Table 2:** The prognostic factors affecting progression-free survival (PFS) of patients at ≤65 years old.

PFS	Univariate Analysis			Multivariate Analysis		
	HR	95% Cl	*p* Value	HR	95% Cl	*p* Value
Ig A subtype	1.059	0.418–2.684	0.904	0.468	0.132–1.659	0.240
ISS 1	1.001	0.432–2.310	0.962	1.410	0.452–3.206	0.498
ISS 2	1.047	0.462–2.370	0.913	1.429	0.488–4.185	0.514
ISS 3	0.944	0.420–2.123	0.889	0.784	0.236–2.603	0.691
Hemoglobin < 10 gr/dL	1.817	0.969–3.407	0.062	2.215	1.013–4.844	0.046 *
Creatinine ≥ 2 mg/dL	1.698	0.871–3.310	0.120	1.349	0.523–3.479	0.536
Calcium > 11 mg/dL	2.822	1.426–5.582	0.003 *	0.500	0.183–1.365	0.176
Litic Bone lesions	1.177	0.561–2.469	0.666	1.063	0.395–2.859	0.903
Plasmacytoma	1.714	0.861–3.412	0.125	3.044	1.246–7.437	0.015 *

HR: hazard ratio; ISS: international staging system; PFS: progression-free survival; *: Statistically significant *p*-value.

**Table 3:** Multivariate Cox regression models for progression-free survival (PFS) and overall survival (OS).

Variables	PFS		OS	
	HR (95% CI)	*p* Value	HR (95% CI)	*p* Value
Age	1.01 (0.9–1.05)	0.280	1.01 (0.98–1.05)	0.292
Hemoglobin < 10 gr/dL	0.78 (0.27–1.07)	0.078	0.64 (0.33–1.24)	0.193
Albumin	0.57 (0.34–0.96)	0.035 *	0.48 (0.29–0.79)	0.004 *
ISS Stage	0.4 (0.18–0.89)	0.025 *	0.38 (0.16–0.91)	0.03 *
Litic Bone Lesions	1.05 (0.57–1.91)	0.867	1.27 (0.71–2.26)	0.418
Creatinine ≥ 2 mg/dL	0.95 (0.46–1.96)	0.909	1.40 (0.69–2.85)	0.340
Melphalan dose	0.58 (0.32–1.06)	0.079	0.56 (0.30–1.05)	0.071

OS: overall survival; HR: hazard ratio; PFS: progression-free survival; *: Statistically significant *p*-value; ISS: international staging system.

## Data Availability

Data used in this study are available from the corresponding author upon request.
